# Using omics approaches to understand pulmonary diseases

**DOI:** 10.1186/s12931-017-0631-9

**Published:** 2017-08-03

**Authors:** Mengyuan Kan, Maya Shumyatcher, Blanca E. Himes

**Affiliations:** Department of Biostatistics, Epidemiology and Informatics, University of Pennsylvania, 402 Blockley Hall 423 Guardian Drive, Philadelphia, PA 19104 USA

**Keywords:** Pulmonary diseases, Genomics, Transcriptomics, Epigenomics, Proteomics, Metabolomics

## Abstract

Omics approaches are high-throughput unbiased technologies that provide snapshots of various aspects of biological systems and include: 1) genomics, the measure of DNA variation; 2) transcriptomics, the measure of RNA expression; 3) epigenomics, the measure of DNA alterations not involving sequence variation that influence RNA expression; 4) proteomics, the measure of protein expression or its chemical modifications; and 5) metabolomics, the measure of metabolite levels. Our understanding of pulmonary diseases has increased as a result of applying these omics approaches to characterize patients, uncover mechanisms underlying drug responsiveness, and identify effects of environmental exposures and interventions. As more tissue- and cell-specific omics data is analyzed and integrated for diverse patients under various conditions, there will be increased identification of key mechanisms that underlie pulmonary biological processes, disease endotypes, and novel therapeutics that are efficacious in select individuals. We provide a synopsis of how omics approaches have advanced our understanding of asthma, chronic obstructive pulmonary disease (COPD), acute respiratory distress syndrome (ARDS), idiopathic pulmonary fibrosis (IPF), and pulmonary arterial hypertension (PAH), and we highlight ongoing work that will facilitate pulmonary disease precision medicine.

## Background

**Fig. 1:**
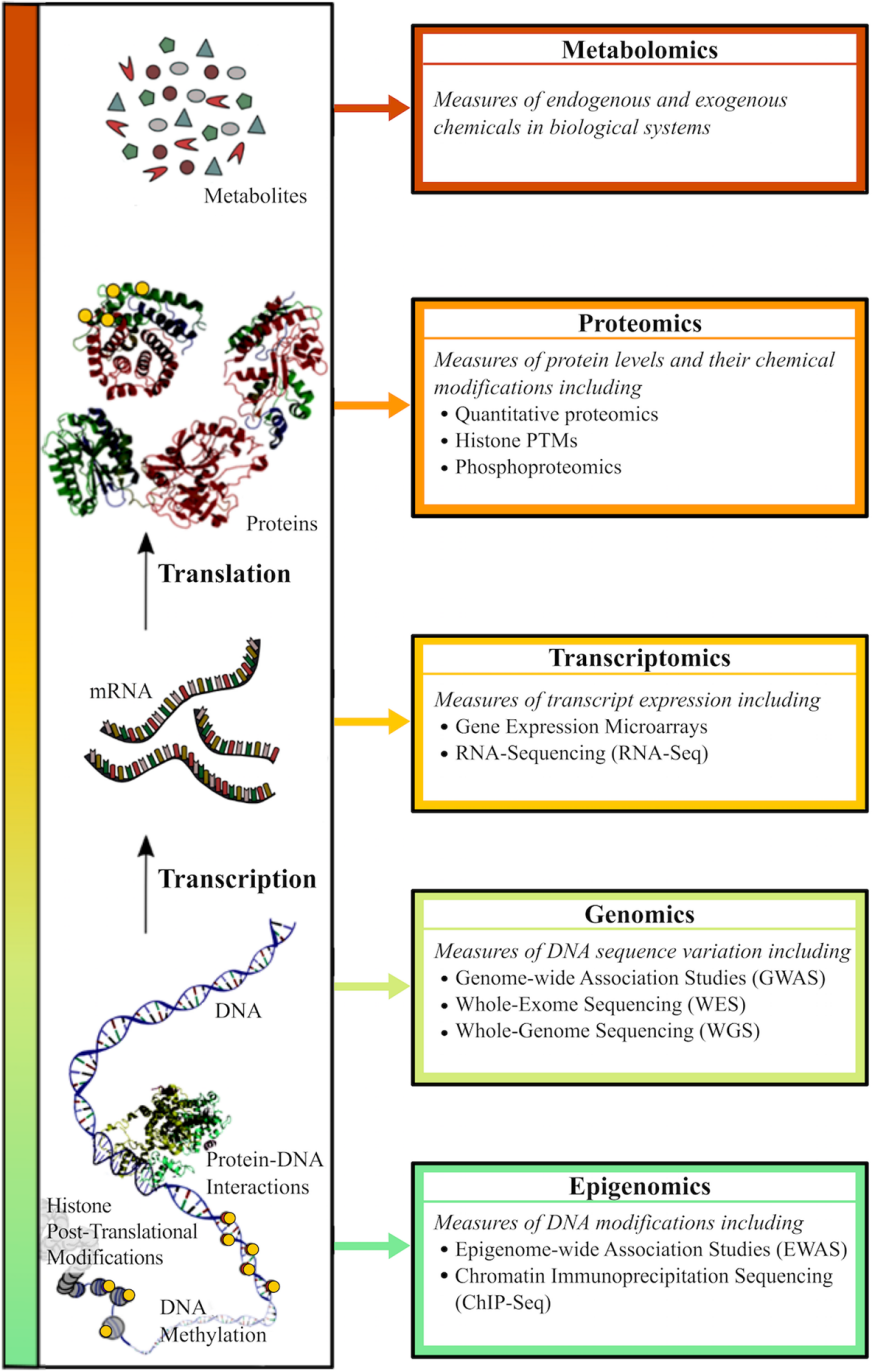
Summary of omics approaches discussed: layers of biological data (*left*) with corresponding omics techniques used for their characterization (*right*)

The commoditization of high-throughput biotechnologies has enabled the collection of an unprecedentedly large number of so-called *omics* datasets by biomedical researchers. Starting with DNA microarrays in the 1990s and expanding to next-generation sequencing (NGS) in the 2000s, omics approaches now capture a wide variety of biological measurements, spanning DNA variation to chemical modifications of proteins [Fig. 1] [1–3]. As the repertoire of available omics approaches continues to expand with the development of methods that combine existing assays and new technologies, an unbiased characterization of biological systems at ever-increasing resolutions is possible [4, 5]. Early successes in the use of omics technologies to understand disease and enable drug development [6, 7] have resulted in optimism that many more effective diagnostic tests and treatments tailored to individuals’ genetic, environmental and lifestyle factors will be developed. The commonplace use of such tests and diagnostics is often referred to as *personalized medicine*, or more recently, *precision medicine* [8, 9].

Decision-making in medical care for decades has often relied on a “one-size-fits-all” approach that applies mean-effect-size results from studies to individual patients [10]. The goal of *precision medicine*, in contrast, is to allow for more accurate treatment and prevention decisions based on matching a patient’s exposure, lifestyle and biological profile to that of similar patients with measured outcomes. The goal of many omics studies thus far has been to build a knowledgebase of omic variation using single-technology approaches that will help enable precision medicine by providing reference data to identify groups of individuals who share various attributes. In addition to collecting omics data, the application of sophisticated algorithms and use of extensive computational resources to integrate datasets are required to fully characterize diverse patients [11, 12].

Here, we provide a broad overview of how omics approaches have been used to understand five complex pulmonary diseases that stand to benefit from personalized diagnostics and treatment:

1. Asthma, an inflammatory disease characterized by variable airflow limitation and airway hyperresponsiveness, that affects over 25 million Americans [13].
2. Chronic obstructive pulmonary disease (COPD), a disease characterized by alveolar destruction, shortness of breath, cough, and sputum production, which is estimated to affect over 24 million Americans [14].
3. Acute respiratory distress syndrome (ARDS), a severe lung condition with high fatality rate that typically occurs in critically ill patients and is characterized by the acute development of diffuse alveolar injury leading to respiratory failure [15].
4. Idiopathic pulmonary fibrosis (IPF), a progressive disease that is the most common and lethal type of idiopathic interstitial pneumonias and is characterized by scarring fibrosis with an unpredictable course [16].
5. Pulmonary arterial hypertension (PAH), a disease that predominantly affects women and is characterized by endothelial proliferation and smooth muscle hypertrophy of small pulmonary arteries, in situ thrombosis, and plexiform lesions that lead to right ventricular failure, and ultimately, death [17].

Some of the main findings obtained by applying omics approaches to these diseases are summarized in Table 1. As our goal is to offer an extensive multi-level omics review, readers interested in learning more about specific techniques or their application to disease are encouraged to read the papers in Table 1 and other in-depth resources.

**Table 1:** Summary of main findings for various omics approaches applied to the study of pulmonary diseases

Disease	Main findings
Genomics	
Genome-wide/Exome-wide microarray	
Asthma	Prominent asthma-associated loci are 17q21 locus (including *ORMDL3, GSDMB*), *IL33*, *IL1RL1, TSLP* [32] Rare, potentially functional variants within *GRASP*, *GSDMB*, and *MTHFR* are associated differently with asthma in subjects of Latino and African ancestry [56] Severe asthma-associated loci are *CDHR3*, *GSDMB*, *IL33* and *IL1RL1* [43]
IgE levels	*FCER1A* and *HLA-DQB1* are associated with IgE levels, the latter in asthma patients only [47]
Asthma drug response	*SPATS2L* is associated with bronchodilator response in asthma patients [54] *GLCCI1* is associated with lung function in patients treated with inhaled glucocorticoids [55]
COPD	Robust COPD-associated loci are *FAM13A*, *CHRNA3*/*CHRNA5*/*IREB2*, *HHIP* [33] Rare, potentially functional variants in *MOCS3*, *IFIT3* and *SERPINA12* are associated with COPD and airflow limitation [58]
COPD endotype	*BICD1* is associated with emphysema [44]
Lung function	*FAM13A*, *HHIP, HTR4* are associated with both lung function (i.e. FEV1 and FEV1/FVC ratio) and COPD [48]
IPF	*TERT* and *MUC5B* are associated with IPF [61, 62]
PAH	*CBLN2* is associated with PAH in patients without *BMPR2* mutations [65]
Whole exome sequencing	
COPD	Increased number of rare, non-silent mutations in *DNAH8*, *ALCAM*, *RARS*, and *GBF1* are present in severe, early-onset COPD [57]
PAH	High penetrance missense variants in *KCNK3* and *TOPBP1* found in familial PAH and idiopathic PAH [67, 68]
Transcriptomics	
Gene expression microarray	
Asthma	Bitter taste receptors have increased expression in severe asthma [86] Distinct epithelial gene expression signature involving in interferon response found in severe childhood asthma [87] Transcriptional activation of circulating CD8+ T cells but not CD4+ T cells present in severe asthma [88]
Asthma endotype	Severe asthma subgroups defined based on transcriptomic and clinical characteristics [92–94]
Asthma drug response	*KLF15* is a glucocorticoid responsive gene in ASM cells [101]
COPD	Distinct PBMC gene expression representing immune, inflammatory response and sphingolipid metabolism pathways, and including *ASAH1,* involved in COPD and emphysema [97] Sputum gene expression changes, including *IL18R1,* are associated with COPD severity [98] Increased gene expression of neutrophil proteases found in COPD patients with respiratory distress [99]
ARDS	Blood neutrophil-related genes and pre-elafin are potential biomarkers in early sepsis-induced ARDS [106] and in acute stage of ARDS [107], respectively Neutrophil gene expression changes in ARDS similar to those in sepsis and burns [108]
IPF	*CCNA2* and alpha-defensin genes are upregulated in lung tissue of IPF patients with acute exacerbations [109] PBMC *CD28*, *ICOS*, *LCK*, and *ITK* are predictors of poor outcomes (transplantation, death) in IPF [110]
PAH	Expression changes in *BMP2* and *BMPR2* are associated with PAH, even in tissues from patients without *BMPR2* mutations [114]
RNA-Seq	
Asthma	Differential expression of *SLC26A4*, *POSTN*, and *BCL2* observed in endobronchial biopsies from asthma patients [89]
Asthma drug response	*CRISPLD2* is a glucocorticoid responsive gene in ASM cells [103] Glucocorticoid-induced genes in ASM from asthma donors include *FAM129A* and *SYNPO2* [104] Cytokine gene expression is modulated by vitamin D treatment in ASM [105]
IPF	Splicing changes in lung tissue *COL6A3* and *POSTN* are associated with IPF [111]
Epigenomics	
Methylation microarray	
Asthma	Hypomethylation of *IL13*, *RUNX3* and *TIGIT* observed in PBMCs of patients with persistent atopic asthma [136] *SMAD3* methylation at birth is associated with asthma in children of mothers with asthma [140]
IgE levels	*AFPM1*, *ACOT7*, and *MND1* methylation are associated with total serum IgE levels in Hispanic children [141] Serum IgE levels are associated with low methylated loci within/near genes encoding known eosinophil products (e.g., *IL5RA*, *IL1RL1, GATA1*) [142]
COPD	Methylation of *C10orf11,* a known COPD-associated gene identified via GWAS, observed in lung of smokers who develop COPD [134] *EPAS1* identified as a key regulator of COPD by combining lung methylation and gene expression data [145]
IPF	Methylation changes observed in *CDKN2B*, *CAR10* and *MGMT* in fibroblasts from IPF patients [150] Hypermethylation of *CASZ1,* and subsequent gene expression changes, are observed in lung of IPF patients [153]
ChIP-Seq	
Asthma	H3K4me2-marked enhancers in T cells are enriched for asthma-associated SNPs and Th2 cell type [154]
Asthma drug response	Glucocorticoid receptor and p65 cooperatively regulate anti-inflammatory gene expression in airway epithelial cells [130]
Proteomics	
Asthma	Plasma protein levels of CCL5, HPGDS, NPSR are associated with childhood asthma [162]
COPD	CTSD, DPYSL2, TGM2, and TPP1 are potential COPD biomarkers; TGM2 in induced sputum and plasma is not associated with smoking but is associated with COPD severity [165]
ARDS	Pathways including inflammation and epithelial injury are associated with ARDS but ARDS-specific biomarkers have not yet been identified [167]
IPF	Levels of apolipoprotein A1, hemoglobin α, hemoglobin β [168], pulmonary fibrosis mediators and eosinophil- and neutrophil-derived proteins [169] differ in IPF patients vs. controls
PAH	TCTP is a mediator of endothelial prosurvival and growth signaling in PAH [173]
Metabolomics	
Asthma	Pathways relating to hypoxia response, oxidative stress, immunity, inflammation, lipid metabolism and the tricarboxylic acid cycle were identified as significant in at least two of 21 asthma metabolomics studies. [180]
COPD	Sphingolipids are highly expressed in sputum of smokers with COPD than smokers without COPD [191]
ARDS	Octane, acetaldehyde and 3-methylheptane in exhaled breath discriminate ARDS patients from other intensive care unit patients [194]
ARDS endotype	A subgroup of ARDS patients with 235 overexpressed metabolites in pulmonary edema fluid had higher mortality rate [197]
IPF	Distinct changes observed in IPF lung tissues vs. controls include increased lactic acid [198], and changes in adenosine triphosphate degradation, glycolysis, glutathione biosynthesis, and ornithine aminotransferase pathways [199]
PAH	Decreased arginine and increased nitric oxide was found in PAH lung tissues vs. healthy controls [200]
Integrative Omics	
Asthma	Asthma susceptibility loci are lung eQTLs, including a 17q21 locus associated with *GSDMA* mRNA expression levels. Network analyses of eQTLs and GWAS results identified SOCS3 pathway as a key driver of asthma [209]
COPD	eQTLs near previously reported COPD GWAS loci (*FAM13A*, *CHRNA3/5, HHIP*) help identify potential functional loci [210] COPD blood pQTLs for surfactant protein D, vitamin D binding protein, and TNFRSF10C are associated with COPD phenotypes; association between eQTLs and pQTLs was low [211]
Single Cell RNA-Seq	
IPF	Coexpression of different cell-specific markers in IPF cells demonstrating “Indeterminate” states of differentiation in IPF [224]

### Genomics

DNA sequence variation is known to cause, or confer risk for, various rare and common diseases, and genetic testing is increasingly being integrated into medical practice [18, 19]. Starting in 2005, as initial cataloging efforts of common DNA single nucleotide polymorphisms (SNPs) led to the design of commercially available microarrays [20], many investigators sought to relate SNPs to disease presence via genome-wide association studies (GWAS). Since then, over 2600 GWAS have been completed for a wide range of phenotypes [21]. With the advent of NGS in the late 2000s, attempts to relate DNA sequence variants to diseases have extended to whole-exome and whole-genome sequencing [22]. Because an individual’s DNA sequence in non-cancerous somatic cells is relatively stable over time and equal in all such cells, obtaining high quality DNA via a single peripheral blood or saliva sample at any point in a person’s life can be used to measure an individual’s sequence variation across the genome [23]. The availability of cost-effective technology, ease of sample collection, and commonality and stability of DNA sequence across a person’s cells and lifetime, have resulted in genomics studies’ sample sizes outpacing that of all other omics approaches.

#### Genome-wide association studies (GWAS)

Based on the hypothesis that common genetic variation underlies complex disease risk, GWAS statistically evaluate whether the frequency of SNP alleles or genotypes differs between affected and unaffected individuals [24]. Using case/control or family-based designs, current GWAS evaluate differences in more than 1 million common SNPs (i.e., those with minor allele frequency (MAF) ≥5% in a reference population) on microarrays. Because so many significance tests are performed in GWAS, multiple comparisons correction of raw scores is necessary to avoid a high false-positive rate of association findings [25]. A commonly employed threshold for genome-wide significance is a *p*-value <5 × 10^−8^, corresponding to a Bonferroni correction applied to a nominally significant *p*-value of 0.05 for 1,000,000 tests (i.e., =0.05/1,000,000). Although there is some merit to criticizing the inability of GWAS results to explain a large portion of complex disease risk [26], the identification of many common variants with small-to-modest effect sizes but reproducible signals that are leading to clinically useful insights has garnered strong support for GWAS among some researchers [27]. To facilitate the translation of genetic association results into functional insights, GWAS results are provided in the GWAS Catalog [21] and the National Heart, Lung, and Blood Institute (NHLBI)-curated Genome-Wide Repository of Associations between SNPs and Phenotypes (GRASP) [28]. Genotype data itself can be found in the NIH’s database of Genotypes and Phenotypes (dbGaP), which also archives individual-level phenotype, sequence data, and association results provided by investigators [29]. Additionally, the U.K. Biobank has made available to all bona fide health researchers genome-wide genotyping data for over 500,000 U.K. residents, along with in-depth health record and phenotype data [30].

#### Whole-exome sequencing (WES) and whole-genome sequencing (WGS)

As NGS costs decreased, WES, and subsequently WGS, have become preferred technologies to characterize the genome. Compared to genotyping microarrays, both technologies offer the advantage of being able to identify rare and novel variants (typically with MAF <1%–5%) [22]. With WES, sequencing costs are a fraction of WGS ones, as only protein-coding variants are targeted under the rationale that functional rare variants are most likely to be in regions of the genome that are translated into proteins. Ultimately, WGS will be the preferred method to characterize the genome for its ability to capture all types of sequence variation. Novel statistical approaches have been developed to analyze WES/WGS data in response to the challenges associated with detecting rare variants and measuring their association with diseases. Considerations include proper DNA sequence alignment to have confidence that detected rare variants are not sequencing errors, adequacy of sample sizes, classification of variants by their presumed function, and importantly, availability of computational resources for storing and analyzing large datasets [31]. Because WES/WGS projects for complex diseases require large sample sizes and substantial funding, some of the most notable projects have been coordinated by government agencies. The NHLBI-Exome Sequencing Project (ESP), for instance, used existing disease-specific cohorts to identify rare variants associated with complex diseases, including asthma, COPD, and acute lung injury [31]. The more recent Trans-Omics for Precision Medicine (TOPMed) Program of the NHLBI focuses on obtaining WGS and other omics data for a greater number of existing population-based studies with the goal of enabling precision medicine in heart, lung and blood-related diseases.

#### Genomics of pulmonary diseases

Many asthma and COPD GWAS have been published and thorough reviews have detailed their findings and limitations [32, 33]. The most prominent GWAS of both diseases are large meta-analyses that pool cohorts gathered by investigators around the world and thus have good statistical power to detect associations. Two major published asthma GWAS consortium projects are GABRIEL [34], consisting of European cohorts, and EVE [35], consisting of diverse North American cohorts. Major COPD GWAS include ECLIPSE, COPDGene, and ICGN cohorts [36, 37]. The most well-known and highly replicated asthma association signal is within the 17q21 locus that includes the *ORMDL3* and *GSDMB* genes [34, 35]. Although this signal is specific to childhood-onset asthma, and studies have made some progress in understanding the function of these genes, the precise mechanism by which variants in this region modify asthma risk is unknown [38–40]. Other robust asthma associations have been found in and near *IL33*, *TSLP,* and *IL1RL1* [34, 35], supporting the notion that epithelial cell-derived cytokines play a critical role in promoting the differentiation and activation of T helper 2 (Th2) cells in asthma pathogenesis [32]. Prominent COPD GWAS loci include *FAM13A*, *CHRNA3*/*CHRNA*5/*IREB2,* and *HHIP* [33]; functional work to understand the mechanisms by which they modulate COPD risk in ongoing. Notably, *HHIP* haploinsufficiency has been found to result in increased age-related emphysema in a mouse model [41], and variation in the *CHRNA3*/*CHRNA*5 locus is associated with nicotine dependence and lung function, suggesting its role in COPD is related to tobacco use and/or metabolism [42].

Due to the complex nature of asthma and COPD, there have been attempts to increase power to detect gene associations and clarify their functional role by measuring associations with objective secondary quantitative phenotypes and specific disease endotypes. This precise approach has been successful in some cases. A severe asthma GWAS identified a novel locus, *CDHR3*, that had not been observed using broader asthma definitions, in addition to the known asthma susceptibility loci *GSDMB*, *IL33,* and *IL1RL1* [43]. In COPD, SNPs at the *BICD1* gene were uniquely associated with emphysema [44]. GWAS has been used to study levels of IgE, an antibody that mediates allergic diseases and is elevated in some asthma cases [45, 46]. Some associations found correspond to IgE levels broadly (e.g., *FCER1A*), while other others (e.g., *HLA-DQB1*) appear to be specific to elevated IgE among asthma patients [47]. GWAS of forced expiratory volume in one second (FEV_1_) and FEV_1_-to-forced vital capacity (FVC) ratio have found that SNPs in genes modifying lung function in healthy adults overlap with some of those that confer COPD risk, including *FAM13A*, *HHIP,* and *HTR4,* suggesting that these genes modulate COPD risk via changes in lung function [48].

Bronchodilator and glucocorticoid medications are common drugs used in the treatment of asthma and COPD [49, 50]. Inhaled short acting bronchodilators (i.e., β_2_-agonists for asthma and COPD, anticholinergics for COPD) are used to provide quick symptom relief [49], while inhaled glucocorticoids are anti-inflammatory controller medications that decrease symptoms with regular use [49, 51]. Patients respond differently to these and other asthma and COPD medications, and there is evidence that genetics plays a role in determining drug response [52, 53]. While efforts to develop pharmacogenetic tests for asthma and COPD drugs have not moved beyond early stages, GWAS of pharmacogenetic traits have found novel gene associations. For example, *SPATS2L* has been associated with bronchodilator response [54], and *GLCCI1* has been associated with lung function outcomes in asthma patients who used inhaled glucocorticoids [55].

Rare variants do not appear to confer much risk for asthma or COPD based on studies published thus far, although future efforts with larger and precisely phenotyped subjects may yield more promising results. One exome study found some evidence of population-specific low-frequency variants being associated with asthma in the following genes: *GRASP* and *GSDMB* among Latinos, and *MTHFR* among African Americans/African Caribbeans [56]. Findings from COPD exome studies have provided some evidence of rare variant association in novel and previously reported regions, but most results do not meet exome-wide statistical significance levels [57, 58]. Thus, rare variants are unlikely to account for a significant proportion of asthma or COPD heritability.

Compared to asthma and COPD, fewer GWAS have been published for ARDS, IPF and PAH, in large part due to their lower prevalence, which results in difficulty recruiting cohorts of sample size necessary to detect statistically significant associations. An ongoing ARDS GWAS by the iSPAAR Consortium has reported in abstract form that moderate ARDS association signals are found in *FARP1* [59, 60]. IPF GWAS performed in Japanese and European individuals have identified genetic risk loci within *TERT*, *TOLLIP*/*MUC5B,* and *SPPL2C* [61, 62]; a GWAS of the broader phenotype idiopathic interstitial pneumonia identified an association within the *MUC5B* promoter that was also associated with IPF [63]. One form of PAH, familial PAH, is known to be caused by genetic mutations, especially in the *BMPR2* gene [64], indicating that genetic loci may confer risk to other forms of PAH. Indeed, a PAH GWAS using cases without *BMPR2* mutations detected an association near *CBLN2* with an odds ratio of 1.97 (95% CI: 1.59–2.45) and *p*-value 7.5 × 10^−10^, suggesting that other loci might be identified via future GWAS [65]. As expected for rare diseases, in which rare loci of larger effect size may modulate disease risk in a small number of individuals, WES has been used to identify high penetrance rare variants in both familial and idiopathic PAH and in familial IPF [66–69], suggesting that there is promise in continuing to apply WGS to understand PAH and IPF.

### Transcriptomics

Characterizing a transcriptome, or all transcripts expressed in a cell or tissue, entails capturing a static measure of a dynamic process that depends on many factors, including developmental stage, health status, time of day, and recent exposures [70]. As such, the goal of most transcriptomic studies is to compare cells or tissues under controlled conditions or disease states to identify major changes in gene or transcript expression that lead to specific functional hypotheses or biomarker development. In parallel with genomics, transcriptomes were first characterized using microarrays, and shortly after the advent of NGS, their measure was extended to RNA-Seq. Although microarrays have remained a cost-effective and widely used technique [71], a primary microarray supplier (Illumina, Inc., La Jolla, CA) discontinued its expression arrays in Dec. 2016, suggesting that sequencing will soon replace arrays entirely.

Because cell and tissue types have their own characteristic transcriptomes [72], selecting the proper site to collect RNA for a study is critical. For pulmonary disease transcriptomic studies, RNA is often extracted from 1) blood, either as whole blood, peripheral blood mononuclear cells (PBMCs), neutrophils, CD4+ T cells, or CD8+ T cells; 2) lung and airway tissues, either as whole lung, endobronchial biopsies, airway smooth muscle cells, or bronchial epithelial cells; 3) induced sputum; and 4) bronchoalveolar or nasal lavage fluids. Several disease-relevant genes and pathways have been identified via transcriptomic studies, and transcriptomics results have been used to identify disease sub-phenotypes – so called *endotypes* [73]. Although most results from transcriptomics have not yet yielded diagnostic tests or new drugs for the five diseases discussed, given the growing sample size and careful design of ongoing transcriptomic studies, translation of more results into actionable clinical insights may occur in the near future.

#### Gene expression microarrays

Since 1995, gene expression microarrays, which are designed based on known transcripts, have been the primary technique used for gene expression profiling. Recent human mRNA arrays measure expression of all known genes with over 40,000 probes, and additional platforms are available to measure expression of micro RNA (miRNA) and long-non-coding RNA (lnRNA). Analysis of microarrays required the development of new methods, most of which are now well established and readily accessible to investigators with various analytical backgrounds [74]. As with GWAS, multiple comparisons correction procedures are necessary to reduce false-positive findings, given the large number of tests performed. Public repositories such as Gene Expression Omnibus (GEO) that host transcriptome data of over 140,000 assays [75], have facilitated re-use of gene expression data for various purposes, including increasing transparency and ensuring reproducibility of published findings [76].

#### RNA-Seq

RNA-Seq allows for sequencing and quantification of transcripts in a cell or tissue at unprecedented depth [77]. Compared to microarrays, RNA-Seq is able to (1) quantify a greater portion of RNA, (2) quantify RNA at baseline, rather than only measure fold changes across conditions, and (3) cover a wider dynamic range of signal [78]. Although RNA-Seq has been shown to provide accurate and reproducible results [79], controversy about the best way to analyze data still exists and development of related methods is ongoing [80]. Along with microarray data, publicly available RNA-Seq data can be found via GEO, although RNA-Seq data is hosted in the Sequence Read Archive (SRA) along with other sequencing data [81].

#### Transcriptomics of pulmonary diseases

Many transcriptomic studies of asthma and COPD have been performed, with studies increasing in sample size and including a wider range of cell and tissue types over the past 10 years [82–84]. Overall, such studies have found a lot of heterogeneity in expression patterns among patients and no clear expression signature that distinguishes patients from healthy controls. Such observations have led to studies that are more restrictive in their definition of asthma or COPD, and to studies that attempt to use expression patterns to identify disease endotypes using unbiased analytic approaches [85].

One prominent asthma study compared expression profiles of white blood cells from 17 severe asthma patients, 19 well-controlled asthma patients, and 18 healthy controls and identified bitter taste transduction receptors (*TAS2Rs*) as highly expressed in severe asthma [86]. Due to experimental evidence showing that *TAS2Rs* are bronchodilators and reduce inflammation, *TAS2Rs* are now candidate drug targets in asthma. Other microarray studies have found that (1) genes involved in interferon response, including *GSDMB*, one of the genes in the 17q21 locus whose variants are strongly associated with asthma, distinguish severe asthma epithelial cells [87], and (2) CD8+, but not CD4+, T cells have gene expression changes that distinguish severe asthma vs. mild asthma [88]. An RNA-Seq study that compared transcriptome profiles of endobronchial biopsies from asthma patients vs. controls identified 46 differentially expressed genes, including *SLC26A4*, *POSTN*, and *BCL2*, but these results have not been further validated [89].

Transcriptomic data has been used, in combination with clinical variables, to identify asthma endotypes by utilizing unsupervised algorithms to identify expression signatures that characterize groups of patients [90–94]. The Unbiased Biomarkers in Prediction of Respiratory Disease Outcomes (U-BIOPRED) Study Group found that peripheral blood of patients with severe asthma could be divided into groups according to differential response to oral steroids [92]. Another U-BIOPRED study based on sputum transcriptomics found that patients with severe asthma could be clustered into four stable groups with distinct clinical characteristics (i.e., well-controlled moderate-to-severe asthma; late-onset severe asthma with a history of smoking and chronic airflow obstruction; late-onset severe asthma in non-smokers with chronic airflow obstruction; obese female patients with uncontrolled severe asthma and normal lung function) [93]. The genes defining such groups reflect distinct molecular mechanisms of disease and thus may lead to biomarkers and group-specific treatments. The Severe Asthma Research Program (SARP) has also sought to identify asthma endotypes, most recently finding via a weighted gene coexpression network analysis that while they could not selectively identify severe asthma patients, genes in network modules linked to epithelial growth and repair and neuronal function were markedly decreased in severe asthma [94].

While in vitro exposure to tobacco produces a strong gene expression signature [95] and some of these changes may persist in lung tissue of past smokers [96], a broad gene expression signature for COPD has not been found and may not exist, given that COPD can occur via different physiological processes. One study comparing PBMC expression changes in past/current smokers with and without COPD and emphysema found 26 genes that distinguished those with disease, representing immune and inflammatory responses as well as sphingolipid metabolism [97]. Other human transcriptomic studies of COPD have searched for differences in expression among COPD patients. One large study of sputum from 148 COPD patients found gene expression changes that characterize the extent of emphysema and airflow limitation, including in *IL18R*, which was also found to have increased protein expression in airway macrophages [98]. Another study found that neutrophil proteases have increased expression in COPD patients with respiratory distress [99]. Beyond COPD alone, comparison of transcriptomic changes in COPD and IPF vs. normal lungs found that both diseases were characterized by increased expression of genes in the p53/hypoxia pathway, suggesting that they share some expression changes that reflect overlapping biological processes [100].

Transcriptome profiling also been used to understand asthma and COPD drug responses. For example, papers describing glucocorticoid-induced gene expression changes have strong and consistent results, largely because the mechanism of action of glucocorticoids includes direct modulation of gene transcription within cell nuclei [101–104]. An early microarray study that investigated the effects of the glucocorticoid dexamethasone treatment on airway smooth muscle focused on functional validation of *KLF15*, which was identified as a novel modulator of airway hyperresponsiveness and has been the focus of various studies since its discovery [101]. RNA-Seq studies to identify transcriptomic differences in donor-derived primary airway smooth muscle cells that were untreated vs. treated with a glucocorticoid have identified other novel glucocorticoid response genes, such as *CRISPLD2* [103]. The effects of a 2-week course of oral prednisolone on gene expression in patients with mild asthma, using airway smooth muscle extracted via laser caption microdissection from bronchoscopy samples, was investigated with RNA-Seq [104]. Comparing samples from 6 patients assigned to glucocorticoid treatment vs. 6 patients assigned to placebo, this study found that 15 genes were significantly differentially expressed between groups, and 2 of the 15 genes, *FAM129A* and *SYNPO2*, were also associated with airway hyperresponsiveness. Studies have also found that airway smooth muscle treated with vitamin D influences cytokine gene expression [105]. In contrast, transcriptomic studies of β_2_-agonist treatment response do not have as strong results, likely because their mechanism of action does not involve direct modulation of gene transcription [103, 105].

As is the case for genomics, fewer ARDS, IPF and PAH transcriptomic studies have been published compared to those of asthma and COPD. ARDS studies indicate that genes related to neutrophil response to infection are modulated in sepsis patients who develop ARDS, as well as during the acute vs. recovery stages [106, 107]. Additionally, polymorphonuclear leucocytes in ARDS patients have gene expression changes that are very similar to those in sepsis and burns [108]. IPF transcriptomic studies have attempted to find markers of acute exacerbations and poor outcome. Using RNA from lung tissue, *CCNA2* and alpha-defensins were identified as upregulated genes during acute exacerbations [109], while a composite model that included levels of *CD28*, *ICOS*, *LCK*, and *ITK* in PBMCs was identified as a useful predictor of death and transplantation in IPF patients [110]. RNA-Seq analysis of lung tissue changes in IPF found that *COL6A3* and *POSTN* have splicing changes associated with disease [111]. A variety of other IPF-associated expression changes have been identified in lung tissue [112] and peripheral blood [113], suggesting that transcriptomic changes will help identify IPF onset and outcomes. For PAH, over 25 transcriptomic studies have been published, most using RNA from lung homogenate, PBMCs and pulmonary artery smooth muscle cells [114]. Among the most significant findings from these studies are that *BMP2* and its receptor, *BMPR2*, have expression changes associated with PAH. While mutations in *BMPR2* are known to lead to familial PAH, expression changes of this gene and others related to its signaling pathway are present in tissues derived from PAH patients who do not have *BMPR2* mutations [115, 116]. Consistent with what is known about PAH pathophysiology, other expression changes that have been observed include elevated expression of the estrogen receptor 1 (*ESR1*) gene, and genes in pathways related to vascular remodeling [114]. Future studies of PAH gene expression changes that are able to better capture changes unique to pulmonary artery smooth muscle cells may shed further light on specific biological changes that may be targeted by drugs.

### Epigenomics

An epigenome refers to genome modifications that regulate gene expression activity and downstream phenotypes, but do not involve DNA sequence variation per se. Epigenomic information can be heritable, but it also varies considerably according to cell type, developmental stage, and environmental exposures [117]. Most epigenomic studies are designed to address the question of whether epigenome states are different in samples with disease or other phenotype vs. those without it. Commonly studied epigenomic phenomena are DNA methylation and histone modifications. In human methylation studies, “methylation” usually refers to the presence of covalently attached methyl groups to 5′ cytosine DNA positions in CpG dinucleotides, a change that typically represses gene expression [118]. Global profiling of DNA methylation can be achieved using quantitative molecular assays such as bisulfite treatment [119] and methylated DNA immunoprecipitation (MeDIP), followed by genotyping or sequencing of DNA [120]. Although whole-genome bisulfite sequencing (WGBS) is the most accurate way to measure DNA methylation, it is an expensive technique. Hence, the considerably more affordable DNA methylation arrays have been widely used. Histone post-translational modifications (PTMs), including methylation, phosphorylation, acetylation and ubiquitynation that occur on N-terminal tails of histones, modulate gene expression by affecting chromatin condensation and the ability of transcriptional proteins to access DNA [121]. To create global maps of genomic locations where histones with modifications of a specific type are present, chromatin immunoprecipitation (ChIP) to select DNA bound to histones with specific modifications is used followed by microarray analysis [120] or sequencing (ChIP-Seq) [122]. More broadly, techniques such as ChIP-Seq can be used to characterize the cistrome, that is, the genome-wide catalog of all short DNA sequences where a transcription factor binds [123]. Because epigenomes are cell-specific, pulmonary epigenomic studies use DNA extracted from disease-related tissues and cells, including blood, PBMCs, lung, and airway. While epigenomic findings related to most pulmonary diseases have not yet yielded diagnostic tests or therapies that are used in clinical practice, some of the modifications identified have provided insights into disease mechanisms that with further study may yield actionable insights.

#### Epigenome-wide association studies (EWAS)

Akin to the concept of GWAS, epigenome-wide association studies (EWAS) are unbiased studies that measure associations between epigenetic modifications across the genome with diseases or phenotypes [124]. To date, EWAS have typically focused on DNA methylation due to the availability of affordable methylation microarrays, including Illumina’s HumanMethylation450 BeadChip, which contains >450,000 probes and, more recently, the MethylationEPIC BeadChip, which contains >850,000 probes [125]. Important considerations related to EWAS design and interpretation include having appropriate sample sizes, accounting for cellular heterogeneity, and noting that causality cannot be inferred from association results [126]. The GEO and SRA resources mentioned earlier host hundreds of methylation datasets obtained via microarray and high throughput sequencing, respectively [127].

#### Chromatin Immunoprecipitation sequencing (ChIP-Seq)

Experimental and analytical protocols for ChIP-Seq have matured as this technique was used for hundreds of Encyclopedia of DNA Elements (ENCODE) experiments, resulting in evidence-based published standards and guidelines [128, 129]. Although ENCODE assayed 118 cell types, neither this consortium nor others have published many ChIP-Seq results specific to cells that are key to pulmonary phenotypes. ChIP-Seq has been used, however, to study gene regulation mechanisms of important pulmonary disease-related drugs that affect gene transcription. The global characterization of NF-κB and glucocorticoid receptor cistromes using ChIP-Seq, for example, has provided insights into how glucocorticoids alter immune response [130]. Recent studies that measure transcription factor binding sites employ more direct techniques, such as Assay for Transposase-Accessible Chromatin using Sequencing (ATAC-Seq). Unlike ChIP-Seq, which indirectly measures chromatin structure via overlapping histone tail modifications, ATAC-Seq probes open chromatin and provides increased resolution of binding sites [131]. ChIP-Seq data for various studies can be found in GEO and SRA, while results for some, most notably those from ENCODE, are available in the UCSC genome browser [127, 132].

#### Epigenomics of pulmonary diseases

Epigenomic studies of asthma and COPD mostly consist of case/control designs that used arrays to measure genome-wide methylation changes [133], with recent studies simultaneously measuring gene expression profiles to more directly link methylation status with gene expression levels [134–136]. One study comparing PBMC methylation changes of 97 inner-city children with persistent atopic asthma to 97 healthy controls found 81 differentially methylated regions, including hypomethylated immune genes in asthma (i.E. *il13*, *RUNX3, TIGIT*) [136]. Analyses of methylation status of 2484 genes that were also correlated with expression levels led to the identification of asthma-associated CpG markers in *RUNX3, IL4,* and *CAT* [136].

Exposures to environmental factors, such as air pollution and maternal tobacco smoking during pregnancy, have been associated with asthma development and exacerbation in late childhood via alterations of DNA methylation [137–139]. A recent EWAS comparing methylation status in cord blood mononuclear cells from 36 children born to mothers with asthma, of whom 18 did and 18 did not develop asthma by age 9 years, identified 589 differentially methylated regions, including one near the asthma-associated gene *SMAD3*, implying that epigenetic changes may contribute to asthma pathogenesis long before disease develops [140].

Total serum IgE EWAS conducted in asthma cohorts found that methylation status of some genes was associated with total IgE levels [136]. In Hispanic children, *AFPM1, ACOT7,* and *MND1* were associated with total IgE levels in PBMCs [141], while a family-based EWAS of blood leukocytes obtained from European nuclear pedigrees found associations between IgE and low methylation at 36 loci annotated to genes (e.g., *IL5RA*, *IL1RL1, GATA1*) encoding known eosinophil products and phospholipid inflammatory mediators. Loci within genes encoding eosinophil products were also found to be hypomethylated in eosinophils of asthmatics with high IgE levels vs. asthmatics with low IgE levels and controls, suggesting potential targets for asthma patient stratification [142].

Epigenomic studies of COPD have identified methylation changes associated with COPD severity, decreased lung function, and systemic glucocorticoid use [143, 144]. Combined analysis of genome-wide DNA methylation and gene expression data in lung tissues obtained from patients with COPD vs. healthy controls identified *EPAS1* as a key regulator of COPD pathogenesis that has been confirmed via functional studies [145]. Because tobacco smoking alters DNA methylation in cells/tissues [146], with changes that persist after cessation [147], epigenomic studies of COPD match cases and controls by smoking status. DNA methylation changes associated with the development of COPD among smokers were observed in airway epithelial cells and lung tissues [134, 135]. The loci involved were enriched for transcription factors and overlapped with known COPD GWAS hits, such as *C10orf11* [134]. Changes in methylation among COPD patients have also been linked to the development of lung cancer, as tumors from non-small-cell patients with COPD had more methylated *CCDC37* and *MAP1B* promoters than those of patients without COPD [148].

An ARDS genome-wide methylation profiling study of whole blood from 114 intensive care unit patients, 39 of whom developed ARDS, used a candidate-gene approach to determine that methylation changes in *MYLK* were associated with genetic variation and modified by ethnicity between ARDS cases and intensive care unit controls [149]. IPF EWAS conducted using lung tissues and fibroblasts have provided preliminary evidence that widespread DNA methylation changes were present in IPF, including in *CDKN2B*, *CARD10*, and *MGMT*, genes that were also differentially expressed at the mRNA and protein levels [150–152]. Combined genome-wide DNA methylation and gene expression data of lung tissues from 94 IPF patients vs. 67 controls identified 738 differentially methylated regions with significant changes in gene expression, also suggesting that DNA methylation changes affecting gene expression contribute to the pathogenesis of IPF, although precise mechanisms of this change are unknown [153]. Genome-wide methylation studies of PAH have not yet been published.

Beyond the ENCODE datasets described above, two salient pulmonary-disease related ChIP-Seq studies have been published. A ChIP-Seq study of H3K4me2 in naïve and memory CD4+ T cells obtained from 12 asthma patients and 12 healthy controls found that H3K4me2-marked enhancers were associated with both asthma susceptibility and Th2 cell type, and that asthma GWAS SNPs were enriched in the Th2 enhancers [154]. Secondly, ChIP-Seq data for glucocorticoid receptor (GR) and NF-κB (p65) in Beas-2B bronchial epithelial cells treated with the glucocorticoid dexamethasone found that GR and p65 cooperatively regulate the expression of anti-inflammatory genes [130].

### Proteomics

Protein expression levels reflect the metabolic state of, and physical processes experienced by, cells. In addition to measures of protein levels themselves, assays to measure critical aspects of protein function, including localization, protein-protein interactions, and post-translational modifications (PTMs) have also been developed. Characterizing the full proteome is still challenging, but technological innovations are improving our ability to obtain cross-sectional time and space snapshots of protein levels. These snapshots reflect observed phenotypes more closely than those of genomic, transcriptomic, or epigenomic techniques [3]. Protein microarrays designed based on known proteins or peptides were the first to increase high-throughput capacity to discover protein biomarkers, analogous to the microarray technologies that were first used to measure global gene expression and variation [155]. Beside array-based assays, high-throughput technologies for proteomics include mass spectrometry (MS)-based techniques (e.g., tandem-MS (MS/MS)) and gel-based techniques (e.g., differential in-gel electrophoresis (DIGE)) [3].

Pulmonary disease proteomics studies have been carried out using induced sputum, pulmonary epithelial lining fluid, bronchoalveolar and nasal lavage fluids, exhaled breath condensate, and blood plasma and serum [156]. Most proteomic studies of pulmonary diseases have case-control designs, with sample sizes ranging from tens to hundreds of samples [156]. While some of the statistical issues relevant to other omics techniques apply to proteomics, the technologies used for proteomics are quite different than those used for next-generation sequencing and arrays, necessitating tailored analytical approaches and introducing new limitations. Additionally, the greater diversity of technologies used to obtain proteomics data has resulted in a slower adoption of standards to identify and report findings. While the creation and use of a public repository analogous to GEO or SRA to store and catalog proteomics data contributed by researchers has lagged relative to other omics data types, the European Bioinformatics Institute’s PRoteomics IDEntifications (PRIDE) database, a standards-compliant repository that now contains data from over 70,000 assays, is a comprehensive and widely utilized resource [157].

#### Quantitative proteomics

Although MS-based approaches have been widely used since the 1980s, recent advances referred to as “next-generation proteomics,” allow for the quantitative characterization of nearly complete proteomes [3]. These newer approaches involve pre-fractionating protein samples or enriching specific subpopulations of peptides (e.g., by selecting for PTMs), and then using liquid chromatography (LC)-MS, which consists of measuring peptides via MS/MS within fractionated portions separated by LC. After MS/MS spectra are converted into corresponding peptide sequences via comparison to a known database, peptides are assembled into proteins [3]. The ProteomicsDB resource contains a draft of the human proteome, consisting of 18,097 human protein-coding genes, identified via the collection of MS-based assays [158].

#### Histone post-translational modifications (PTMs)

In addition to characterizing DNA binding sites for specific histone PTMs via ChIP-Seq, recent high-throughput quantitative proteomics approaches can measure modified forms of histone amino terminal tails using online LC-MS. Via these state-of-the-art techniques, both single and combinatorial histone codes can be interrogated to quantify global changes in PTMs under different conditions [159]. Because analyses of histone PTMs have revealed that a large number of modified residues in histones act as “histone codes” that are associated with specific physiological processes, identifying combinations of PTMs provides a more complete view of how histone states influence gene transcription and lead to specific physiological processes or disease states [160]. Following the identification of histone PTMs that are associated with an outcome, ChIP-Seq can be used to identify specific genes whose expression is altered by the PTMs.

#### Phosphoproteomics

Changing the catalytic activity of proteins via phosphorylation is a ubiquitous mechanism used to control many biological pathways. Over 30,000 phosphorylation sites have been quantitatively identified via MS-based technology, and it is estimated that an additional 500,000 phosphorylation sites exist in the proteome [3]. Phosphoproteomics is a technique that attempts to quantify levels of all phosphorylation sites simultaneously by eluting phosphorylated peptides from solution prior to performing MS. Although phosphoproteomic studies of pulmonary diseases have not been reported so far, this technique has shown promise in other areas. Global phosphoproetomic profiles of thrombin response in human endothelial cells, for instance, were used to identify known and novel phosphorylation sites that may play a role in platelet aggregation [161].

#### Proteomics in pulmonary diseases

A proteomic study of plasma from 106 children with asthma and 68 controls identified three proteins (CCL5, HPGDS, NPSR) that had different plasma levels in asthmatic children compared to controls, suggesting they could be biomarkers [162]. Studies of the induced sputum proteome from asthma and COPD patients vs. healthy controls have found many potential biomarkers, including calgranulin A and B [163, 164]. Comparison of the lung tissue proteome of nonsmokers, smokers, smokers with mild to moderate COPD, and those with severe to very severe COPD found and validated potential COPD biomarkers, including CTSD, DPYSL2, TGM2, and TPP1 [165]. Additionally, increased sputum and plasma levels of TGM2, which were not associated with smoking, were correlated with COPD severity. Although histone PTM studies related to pulmonary diseases have not been published, such studies would provide helpful insights. For example, reduced responsiveness to glucocorticoids in patients with severe asthma and COPD has been attributed to GR modifications mediated via histone deacetylase 2 (HDAC2) [166]. Because HDAC2 modifies histone PTMs widely, understanding its role on a global level would provide a more comprehensive view of how it alters glucocorticoid response.

Proteomics studies have been conducted to find biomarkers for ARDS, and poor outcomes among those who develop it, as the need for drug development and understanding disease pathobiology are particularly high for this disease [167]. Although some pathways have been identified via these studies (e.g., inflammation and epithelial injury), ARDS-specific markers have not yet been identified [167]. Proteomic changes in lung tissue, nasal lavage fluid and bronchoalveolar lavage fluid from patients with IPF compared to healthy controls include differences in apolipoprotein A1, hemoglobin α, and hemoglobin β [168], pulmonary fibrosis mediators (osteopontin, MMP7, CXCL7, CCL18) and eosinophil- and neutrophil-derived proteins [169]. Proteomic changes identified in PAH thus far also suggest proteins (e.g., TCTP) and pathways (e.g., cell contraction, oxidative stress) that may be important, but there are no validated markers to identify patients or subclassify those with PAH [170–174].

### Metabolomics

Metabolites are small molecules (<1 kDa) that participate in chemical reactions within living organisms, and they include endogenous (amino acids, nucleic acids, vitamins) and exogeneous (drugs, toxins) chemicals. Metabolomics refers to the measure of all metabolites in a biological system [175]. Similar to proteomics, metabolomics provides a snapshot of the active physiological status of a cell or tissue. The most common techniques used to characterize the metabolome are nuclear magnetic resonance (NMR) and high-resolution MS [176, 177]. While metabolomics studies are unbiased, the identification of specific metabolites relies on having references to link measured spectra to them. The public repository Human Metabolome Database (HMDB) provides curated metabolomic data, currently listings 42,000 metabolites [178]. This resource allows users to search metabolites by categories (e.g., metabolite name, disease, and biofluid), and it expedites the process of mining metabolomic data. A wide range of biological samples have been used for pulmonary disease metabolomics studies, including blood serum and plasma, induced sputum, exhaled breath condensate, bronchoalveolar lavage fluid, and lung tissue [179]. Due to the non-invasive and convenient nature of acquiring urine in a clinical setting, urine is also sought as a target to identify metabolomics biomarkers.

#### Metabolomics in pulmonary diseases

Metabolomics studies related to asthma have focused on differences between patients and healthy controls, and between those who are responsive to glucocorticoid treatment vs. those who have low responsiveness [180–182]. Despite small sample sizes, varied biospecimens, profliling technologies, and populations, there were consistencies across 21 asthma biomarker studies: several identified the metabolites acetate, adenosine, hippurate, succinate, alanine, and threonine as related to asthma, and the pathways hypoxia response, oxidative stress, immunity, inflammation, lipid metabolism, and TCA cycle [180]. Although high accuracy tests based on metabolic markers in exhaled breath condensate, serum, and urine have been proposed for noninvasive asthma diagnostics and therapeutic monitoring, most have not been validated in independent samples [183–186]. For example, a metabolomic study of volatile organic compounds in exhaled breath condensate samples from 63 children with asthma vs. 57 healthy controls found that eight volatile organic compounds could classify children with asthma with a sensitivity of 89% and a specificity of 95% [187].

Metabolomic studies of COPD have identified potential serum/plasma metabolic markers used for early recognition of COPD development and exacerbation, independently of smoking status, with markers consistently representing chronic inflammation and oxidative stress pathways [188–190]. A study that focused on lipidome metabolites, found that sphingolipids were highly expressed in sputum of smokers with COPD compared to smokers without COPD [191]. A subsequent targeted study of plasma sphingolipids, found that two specific subgroups, sphingomyelins and glycosphingolipids, were associated with emphysema and COPD exacerbations, respectively [192]. A study of urine metabolomic data from patients with asthma and COPD created a diagnostic model to discriminate asthma from COPD that had 90% accuracy on the dataset used for model creation [193]. Validation of this and other previously reported metabolomics-based biomarker tests in independent cohorts is necessary to verify their potential clinical utility.

Metabolomics has also been applied to the search for ARDS biomarkers, with reports based on exhaled breath, plasma, and bronchoalveolar lavage fluid that showed promise to discriminate between 1) ARDS patients and healthy controls, 2) ventilated intensive care unit patients who did or did not develop ARDS, and 3) sepsis patients who did or did not develop ARDS [194–196]. A recent study comparing undiluted pulmonary edema fluid in ARDS patients and control patients with hydrostatic pulmonary edema, identified an endotype of ARDS patients based on a signature of 235 overrepresented metabolites that was associated with a higher mortality rate [197]. IPF metabolomics studies comparing lung tissue of IPF patients vs. healthy controls have found increased levels of lactic acid in lung tissue of patients suggesting a pH-dependent TGF-β activation mechanism that drives myofibroblast differentiation in IPF [198], and metabolite signatures involving the pathways adenosine triphosphate degradation, glycolysis, glutathione biosynthesis, and ornithine aminotransferase [199]. A PAH metabolomics study comparing lung tissues of 8 PAH patients vs. 8 healthy controls found disruption of arginine and oxidative pathways in PAH lung tissues, including decreased arginine and increased nitric oxide [200].

### Integrative omics

The integration of diverse omics datasets provides an opportunity to gain insights beyond those that are possible from individual datasets. Thus far, most integrative omics analyses involve the pairing of two data types, but increasingly complex analyses have been conducted as novel methods have been developed [201]. Genome-wide expression quantitative trait loci (eQTL) studies, the most common paired approach, involve the measurement of associations between genetic variants and gene expression levels. An assumption of eQTL studies is that differing levels of mRNA result in different observable phenotypic outcomes. Thus, if a GWAS hit is also an eQTL, one can hypothesize that the SNP modulates an associated phenotype via changes to level of expression of a specific gene. Due to the cell- and exposure-specific nature of gene expression, eQTLs are most relevant to a GWAS if their results were obtained in a disease-relevant context. The most comprehensive eQTL database currently available is the Genotype-Tissue Expression (GTEx) resource, which includes data for 1641 samples across 43 tissues from 175 individuals [202]. Although this resource is helpful, it does not ideal for pulmonary disease studies, as results for lung are derived from lung tissue, rather than cell-specific lung components. The design of eQTL studies has been extended to relate genetic variants to protein and methylation levels, resulting in so-called protein quantitative trait loci (pQTL) [203] and methylation quantitative trait loci (meQTL) [204] studies. Approaches that consider more than two data types often involve multi-staged analyses, where relationships among datasets are sought in a hierarchical fashion [205, 206]. Prior to integration, individual datasets must be analyzed carefully to reduce propagation of errors, as these compound when performing integrated analyses [11]. Additionally, overfitting errors are more prominent because the number of observations in integrative analyses are much greater than the number of individual samples [11].

Most integrative omics studies in pulmonary diseases are eQTL studies [207, 208]. A large-scale asthma eQTL study of 1111 human lung tissues identified an eQTL within *GSDMA* that was a risk allele for asthma in the GABRIEL GWAS study, and created a network of relationships with eQTL and GWAS data that identified SOCS3 as a key asthma pathway [209]. A large COPD eQTL study that used whole blood and sputum gene expression data from 121 ECLIPSE subjects found eQTLs near previously reported GWAS loci (*FAM13A*, *CHRNA3/5, HHIP*), suggesting hypotheses for how association signals are functionally related to COPD [210]. A COPD pQTL study based on expression levels of 88 blood proteins and a COPD GWAS, found 527 pQTLs, including surfactant protein D, vitamin D binding protein, and TNFRSF10C [211]. Despite not using unbiased proteomic measures, this study showed that pQTLs provide helpful functional links that were absent at the level of eQTLs for most of the genes in question. A broader integrative study of longitudinal FEV_1_ in children with asthma combined GWAS, RNA-Seq, and ChIP-Seq data to identify seven nominally significant variants that could be related to this phenotype [212]. Although this study did not fully integrate datasets and it suffered from having a small sample size, it demonstrated that leveraging multiple datasets can lead to helpful disease insights.

Another approach taken in integrative omics studies is to use networks to model higher-order interactions among biological, environmental, and clinical characteristics of patient groups to identify hypotheses regarding disease mechanisms. A common network approach is to represent molecules (e.g. genes, proteins) and diseases as nodes, and assign edges between nodes according to known or measured relationships. From such networks, various structural measures are made, including the identification of the most highly connected nodes (i.e., the *key* nodes) and of modules consisting of highly connected groups of nodes that are thought to participate in specific biological or pathogenic processes or share other commonalities [213, 214]. Such measures are used to prioritize relationships for further validation studies. Network-based analyses focused on transcriptomic data are most common, and have been applied to identify clinically distinct asthma and COPD subgroups [94, 215, 216].

### Single cell approaches

In contrast to the omics techniques already discussed that use cells or tissue in bulk as starting materials, newer assays are enabling the characterization of single cells [217]. Such approaches offer a more thorough understanding of physiological processes, as single-cell resolution omics data enables the characterization of intra-cellular populations, cell states, and cell transitions that are not observable with population-averaged cell data [218, 219]. The workflow for single-cell techniques is similar to that for their bulk counterparts, but with additional key steps to isolate single cells and amplify the genome component of interest [220]. Novel computational and statistical methods have arisen to deal with challenges related to these techniques. For example, analysis of single-cell RNA-Seq (scRNA-Seq) data, the most widely used single cell approach, involves dealing with a large number of undetected transcripts (i.e., an abundance of zeros), increased variability, and multi-modal expression distributions [221].

Although the number of scRNA-Seq studies is quickly expanding, few related to pulmonary diseases have been published. Mouse model scRNA-Seq studies have been used to 1) reconstruct cell lineage hierarchies of lung epithelial cells, unveiling progenitors of two alveolar type cells and novel cell-type specific markers [222], and 2) identify innate lymphoid cell precursor subsets using mouse bone marrow progenitors [223]. An IPF scRNA-Seq study found that IPF cells often coexpressed markers of alveolar type 1, alveolar type 2, and conducting airway cells, and were thus in “indeterminate” states of differentiation, in contrast to normal lung epithelial cells that expressed alveolar type 2 markers [224]. Future single-cell omics studies will be especially helpful to understand cell populations that are drivers of disease and characterize developmental processes.

## Conclusions

A growing number of pulmonary disease omics studies have been reported over the last decade, covering an ever-increasing number of tissues and using newer technologies. Omics studies thus far have led to insights into disease mechanisms and laid a foundation for biomarker and therapeutic discovery, but our ability to generate hypotheses from omics-based studies has quickly outpaced our ability to translate hypotheses into actionable biologic insights. Genomic and transcriptomic studies of asthma, COPD, ARDS, IPF, and PAH, have identified replicable findings that are the subject of ongoing functional validation studies. Prominent genomics results include the 17q21 locus, *HHIP* and *MUC5B* genetic variants that confer risk for asthma, COPD and IPF, respectively, while prominent transcriptomic results include the differential expression of bitter taste receptor and *KLF5* genes observed in asthma that may lead to novel therapeutic targets. Epigenomic studies have identified methylation patterns specific to COPD (e.g., *C10orf11* in lung), asthma (e.g., *IL13*, *RUNX3*, *TIGIT* in PBMCs) and IPF (e.g., *CASZ1* in lung), although much work remains to characterize cell-specific changes and include more ARDS and PAH samples. Relatively few proteomic and metabolomic studies have been published for pulmonary diseases, but sphingolipids are candidate biomarkers for COPD and a combination of exhaled breath condensates, including octane and acetaldehyde, show promise to become ARDS biomarkers. Studies that have identified asthma, COPD and IPF endotypes thus far show promise in our ability to reliably classify diseases using omics data, and such approaches will extend to ARDS and PAH.

As the knowledgebase derived from omics profiles of healthy, diseased and treated cells from diverse donors grows, defining subgroups of patients with distinct molecular and clinical characteristics will facilitate the development of biomarkers to accurately predict whether a patient has or will develop a specific disease type, or whether a patient is responding or will respond to a particular drug or form of therapy. Integrative analyses of omics data that combine a wide variety of data types will help prioritize mechanistic studies to understand the function of various observed relationships, as well as identify the most promising candidates for prospective biomarker trials. Ongoing and future omics studies covering a broader and diverse set of patients and data types and leveraging integrative analytic strategies will accelerate the advance of pulmonary disease precision medicine.

## Abbreviations

ARDS: Acute respiratory distress syndrome
ChIP-Seq: Chromatin Immunoprecipitation Sequencing
COPD: Chronic obstructive pulmonary disease
eQTL: Expression quantitative trait loci
EWAS: Epigenome-wide association studies
GEO: Gene Expression Omnibus
GWAS: Genome-wide association study
IPF: Idiopathic pulmonary fibrosis
LC: Liquid chromatography
MS: Mass spectrometry
NGS: Next-generation sequencing
NHLBI: National Heart, Lung, and Blood Institute
PAH: Pulmonary arterial hypertension
PBMC: Peripheral blood mononuclear cell
pQTL: Protein quantitative trait loci
PTM: Post-translational modification
RNA-Seq: RNA sequencing
scRNA-Seq: Single-cell RNA-Seq
SNP: single nucleotide polymorphisms
SRA: Sequence Read Archive
WES: Whole-exome sequencing
WGS: Whole-genome sequencing
